# 奥希替尼治疗后继发SCLC转化和*ALK*融合的*EGFR*突变型肺腺癌1例

**DOI:** 10.3779/j.issn.1009-3419.2026.101.09

**Published:** 2026-04-20

**Authors:** Liyan LIAO, Xiaohong LI

**Affiliations:** 410011 长沙，中南大学湘雅二医院病理科; Department of Pathology, The Second Xiangya Hospital, Central South University, Changsha 410011, China

**Keywords:** 肺肿瘤, 表皮生长因子受体, 间变性淋巴瘤激酶, 小细胞肺癌转化, 奥希替尼, Lung neoplasms, Epidermal growth factor receptor, Anaplastic lymphoma kinase, Small cell lung cancer transformation, Osimertinib

## Abstract

表皮生长因子受体（epidermal growth factor receptor, EGFR）基因突变是非小细胞肺癌（non-small cell lung cancer, NSCLC）最常见的驱动基因突变。本文报告1例EGFR敏感突变的肺腺癌患者，经奥希替尼一线治疗后，继发小细胞肺癌（small cell lung cancer, SCLC）转化和间变性淋巴瘤激酶（anaplastic lymphoma kinase, ALK）融合。本病例报道奥希替尼治疗后同时发生SCLC转化与ALK融合，提示二者可作为双重耐药机制并存。再次活检联合基因检测对明确耐药机制、指导个体化治疗至关重要。

## Abstract (en)

Epidermal growth factor receptor (EGFR) gene mutations are the most common driver mutations in non-small cell lung cancer (NSCLC). This article reports a case of a patient with EGFR-sensitive mutant lung adenocarcinoma who developed secondary transformation to small cell lung cancer (SCLC) and anaplastic lymphoma kinase (ALK) gene fusion following first-line Osimertinib therapy. This paper reported a case of concurrent SCLC transformation and ALK fusion occurring after Osimertinib treatment, suggesting that the two can coexist as dual drug resistance mechanisms. Re-biopsy combined with genetic testing is essential for identifying drug resistance mechanisms and guiding individualized therapy.

非小细胞肺癌（non-small cell lung cancer, NSCLC）约占肺癌总数的85%；而小细胞肺癌（small cell lung cancer, SCLC）是一种特殊类型的肺癌，约占15%^[1]^。表皮生长因子受体（epidermal growth factor receptor, EGFR）的激活突变是NSCLC最重要的驱动基因突变之一^[2]^，分为经典突变和非经典突变，主要发生于第18-21号外显子。经典突变主要包括第19号外显子的缺失突变（占45%-50%）和第20号外显子的L858R点突变（占35%-40%），对传统的EGFR-酪氨酸激酶抑制剂（EGFR-tyrosine kinase inhibitors, EGFR-TKIs）敏感^[3]^。然而，所有患者最终都会发生耐药，最常见的耐药机制是发生在第20号外显子的T790M点突变，通常发生在EGFR-TKIs治疗后的8-17个月^[4]^。

奥希替尼（Osimertinib）是对EGFR激活突变和EGFR T790M耐药突变位点不可逆抑制的第三代EGFR-TKIs^[4]^。在FLAURA试验^[4]^中，相比其他EGFR-TKIs，奥希替尼在未经治疗的EGFR突变型NSCLC晚期患者中的疗效更优，其中位无进展生存期（progression-free survival, PFS）和总生存期（overall survival, OS）分别达到18.9和38.6个月。与第一、二代EGFR-TKIs相似，奥希替尼亦难以避免获得性耐药的发生，其耐药模式主要分为EGFR通路依赖型与非依赖型两大类^[5]^。其中，非依赖型耐药机制涉及旁路信号异常活化、致癌基因融合以及肿瘤组织学表型转换等多种形式。在接受TKIs靶向治疗后，大约2%的NSCLC患者出现SCLC转化，对患者耐药前的原始标本和耐药后的再次活检肿瘤组织进行基因检测后发现，发生SCLC谱系转化后的肿瘤组织仍保留原始携带的EGFR激活突变^[6]^。另外，致癌基因融合亦是介导奥希替尼获得性耐药的重要分子机制之一，其中间变性淋巴瘤激酶（anaplastic lymphoma kinase, *ALK*）融合在第三代EGFR-TKIs耐药中可能比在第一、二代中更常见^[7]^。本文报道1例两种耐药机制并存的罕见病例：奥希替尼一线治疗后，EGFR L858R突变的肺腺癌并发SCLC转化和*ALK*融合。本文结合病理、影像及下一代测序（next generation sequencing, NGS）结果分析其分子演化与治疗转归，为临床识别罕见耐药模式、优化个体化治疗策略提供参考。

## 1 病例资料

患者女，80岁，不吸烟，于2019年1月在体检中发现左肺上叶后段孤立性结节，不伴有咳嗽、咳痰、发热，未予治疗。2019年10月14日肺部增强计算机断层扫描（computed tomography, CT）（图1）示“左上肺结节有所进展（12.8 mm×12.0 mm），考虑肺癌可能性大”。2019年10月23日，对左上肺结节行经皮穿刺活检，病理诊断：高分化腺癌（图2A）。免疫组化（immunohistochemistry, IHC）显示：甲状腺转录因子-1（thyroid transcription factor-1, TTF-1）、天冬氨酸肽酶A（novel aspartic proteinase A, Napsin A）、细胞角蛋白7（cytokeratin 7, CK7）均呈阳性（图2B-2D）；神经内分泌标志物分化簇56（cluster of differentiation 56, CD56）、嗜铬粒蛋白A（chromogranin A, CgA）和突触素（synaptophysin, Syn）均为阴性。其他IHC指标：CK（+），CK5/6（-），P40（-），程序性细胞死亡蛋白1（programmed cell death 1, PD-1）（-），程序性细胞死亡配体1（programmed cell death ligand 1, PD-L1）肿瘤阳性细胞比例分数（tumor proportion score, TPS）评分<1%，Ki-67（20%+），肿瘤原发灶-淋巴结-转移（tumor-node-metastasis, TNM）分期cT1bN0M0 IA2期。NGS结果示：EGFR基因21号外显子c.2573T>G（p.L858R），变异等位基因频率（variant allele frequency, VAF）为42.5%；视网膜母细胞瘤蛋白1（retinoblastoma protein 1, RB1）基因7号外显子c.706A>T（p.K236*），VAF为15.3%；平滑化受体（smoothened frizzled class receptor, SMO）基因11号外显子c.1906G>T（p.D636Y），VAF为24.6%。肿瘤突变负荷（tumor mutational burden, TMB）值为1.92个突变/Mb，判定为TMB-低（TMB-low, TMB-L）；微卫星不稳定性（microsatellite instability, MSI）评估结果为微卫星稳定型（microsatellite stable, MSS）。

**图1:**
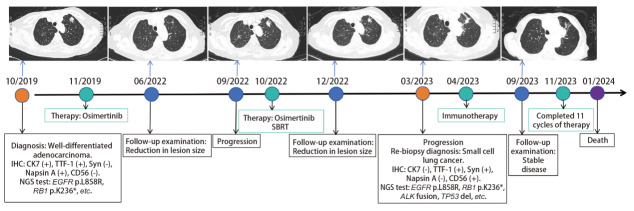
肿瘤在治疗期间重要节点的影像学演变过程。此图展示了不同治疗干预下肿瘤反应的时间演变，并附有组织病理学评估和组织样本的分子生物学检测结果。病理学评估通过HE染色进行形态学观察，随后通过IHC进行确认。基因突变通过NGS分析检测。

**图2:**
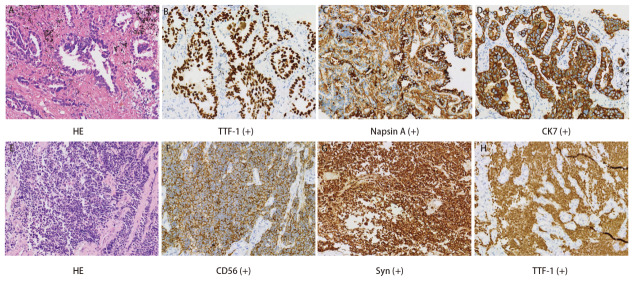
奥希替尼治疗前和治疗后的病理形态学特征。A-D：奥希替尼治疗前，通过HE染色观察肺腺癌的形态学特征（A）；进行IHC染色以观察肿瘤细胞中TTF-1（B）、Napsin A（C）和CK7（D）的表达；E-H：奥希替尼治疗后，通过HE染色观察SCLC的形态学特征（E）；进行IHC染色以观察肿瘤细胞中CD56（F）、Syn（G）和TTF-1（H）的表达。放大倍数：×20。

该患者高龄，美国东部肿瘤协作组体能状态评分3分，拒绝手术治疗。2019年11月1日起口服奥希替尼80 mg、每日1次进行靶向治疗。2022年6月18日复查肺部CT病灶缩小。2022年9月22日肺部CT提示疾病进展（图1）。经多学科诊疗团队讨论后，继续口服奥希替尼，并于2022年10月24日和28日加用左肺结节的体部立体定向放射治疗，50 Gy（单次10 Gy，5次/周）。2022年12月14日复查肺部CT提示左上肺尖后段不规则实性结节较前次检查缩小（图1）。2023年3月3日复查胸部CT提示疾病进展（图1），停用奥希替尼。2023年3月9日，对左上肺结节再次穿刺活检，病理诊断：SCLC（图2E）。IHC示：CD56（+）、Syn（+）、TTF-1（+）（图2F-2H）。其余IHC指标：Napsin A（-）、CgA（-）、CK7（-）、CK（-）、CK5/6（-）、P40（-）、PD-1（-）、PD-L1（TPS评分<1%）、Ki-67（90%+）。NGS检出29个体细胞变异，包括5个单核苷酸变异、23个拷贝数变异（copy number variation, CNV），以及1个融合。部分结果：*EGFR* p.L858R（VAF: 76.2%）、*RB1* p.K236*（VAF: 84.1%）、*SMO* p.D636Y（VAF: 12.4%）、*CRIM1*（intergenic）-*ALK*融合（VAF: 12.3%）、*TP53*缺失（拷贝数：1.0）。TMB为3.84个突变/Mb（TMB-L），MSI状态评估为MSS。

调整治疗策略，2023年3月23日口服依托泊苷胶囊进行化疗。复查血常规示中性粒细胞低至0.53×10^9^/L，血红蛋白低至64 g/L，达到3级骨髓抑制，遂停用依托泊苷胶囊，予以升血小板、白细胞等对症支持治疗。2023年4月17日、5月10日予以第1、2周期免疫维持治疗：阿得贝利单抗1200 mg静脉滴注各1次。2023年5月22日复查胸部CT示：左肺门肿块及纵隔、左肺门淋巴结较前缩小。继续于2023年5月31日、6月21日予以第3、4周期免疫维持治疗。2023年7月12日行第5周期免疫维持治疗：阿得贝利单抗1200 mg单次静脉输注，联合安罗替尼8 mg口服（连用14 d，每日1次）。2023年9月27日复查胸部CT评估疗效（图1），疾病稳定，继续原免疫维持治疗，于11月16日完成第11周期治疗。2023年12月13日复查肺部CT示左肺门区肿块并左肺上叶阻塞性肺不张较前进展，累及邻近食管、胸膜，双侧胸腔新发积液，伴随双肺下叶肺不张表现，心包积液较前增多。对症治疗效果不佳，于2024年1月2日经抢救无效死亡。本例患者接受奥希替尼治疗的PFS达35个月，总用药时长达40个月，免疫治疗的PFS达8个月，总OS达50个月。

## 2 讨论

目前，奥希替尼已成为携带EGFR敏感突变的晚期NSCLC患者一线治疗的首选方案^[8]^。本例患者采用奥希替尼一线治疗，PFS达到35个月，显著长于奥希替尼一线治疗的中位PFS（18.9个月），其潜在原因值得深入探讨。首先，该患者为不吸烟的高龄女性，仅携带单一*EGFR* L858R敏感突变，肿瘤生物学行为通常更依赖EGFR信号通路。其次，肿瘤为高分化腺癌，且TNM分期为早期，说明肿瘤侵袭性相对较低，靶向治疗耐受性更佳。最后，在治疗初期即呈现持续应答，治疗期间并未早期出现*EGFR* C797S突变等常见耐药机制，说明其耐药演变进程相对平缓。该患者出现两种耐药机制并存（SCLC转化和*ALK*融合）。*ALK*等基因融合作为EGFR-TKIs治疗后的罕见旁路激活耐药机制，发生率为3%-10%，在奥希替尼治疗进展的EGFR敏感突变的NSCLC患者中已被多次验证^[5,9,10]^。奥希替尼治疗后出现SCLC转化或获得性ALK融合均有报道，但尚未见两种耐药机制并存的报道，提示该患者耐药机制的复杂性和罕见性。

肺腺癌向SCLC转化在第一代TKIs治疗后属于罕见耐药模式，近年来亦有报道该耐药模式出现在一线或二线应用奥希替尼等第三代EGFR-TKIs治疗后^[5]^。此类转化型SCLC在保留原发驱动基因突变基础上同时获得SCLC特征性分子改变。其中，*TP53*与*RB1*共失活突变是驱动*EGFR*突变型肺腺癌向SCLC转化的核心分子机制^[6]^。本例患者在治疗前已存在*RB1* p.K236*失活突变（VAF: 15.3%），提示肿瘤克隆在初始阶段已具备向神经内分泌表型转化的分子潜能。耐药后该突变VAF显著升高至84.1%，且肿瘤克隆进一步获得*TP53*基因缺失，形成*RB1*/*TP53*双基因失活状态，最终共同驱动SCLC转化。这一分子演化轨迹为本例SCLC转化提供了强有力的分子证据，并提示初诊时*RB1*失活突变可能作为预测SCLC转化风险的潜在独立生物标志物。*EGFR/TP53/RB1*三重突变肺癌患者的全基因组倍增（whole genome doubling, WGD）发生率增高，且SCLC转化后的CNV负荷显著高于初始肺腺癌^[11]^。本例患者的二次活检标本存在大量CNV，符合转化型SCLC的基因突变特征。

TKIs、放疗、化疗均可诱发SCLC转化，而放疗+TKIs联合是最常见的多重压力诱导转化模式^[12]^。局部放疗可诱发肿瘤细胞DNA损伤，并进一步促进基因组不稳定性^[6]^，可能进一步加速染色体重排事件以及神经内分泌通路激活（*RB1/TP53*失活、*PIK3CA-AKT*或*NOTCH*通路异常）的发生，与EGFR-TKIs协同促进SCLC转化。本例患者在奥希替尼靶向治疗联合局部放疗后同步出现SCLC转化与获得性*ALK*融合。结合克隆进化理论，我们推测两种耐药事件并非相互独立，而可能起源于同一肿瘤前体细胞克隆，通过平行克隆进化，同步发生两种耐药机制。一方面，奥希替尼对EGFR信号通路的长期持续抑制作用，形成了较强的治疗选择压力，驱动肿瘤克隆进化，既可诱发神经内分泌分化相关通路激活而导致SCLC转化，也可促使肿瘤细胞通过获得性ALK融合实现旁路激活。另一方面，局部放疗可诱导肿瘤细胞DNA损伤与基因组不稳定性，进一步加速染色体重排事件的发生，并促进肿瘤表型重塑。最终，共同促进SCLC转化与ALK融合双重耐药克隆的出现与富集，形成本例罕见的复杂耐药表型。

发生组织学转化后肿瘤已不再依赖EGFR信号通路，尽管保留了EGFR原有突变，但EGFR蛋白表达下降^[13]^，从EGFR-TKIs治疗中获益有限，故不建议继续应用EGFR-TKIs类药物。值得注意的是，本病例经靶向治疗后出现的转化型SCLC在分子特征、治疗反应及生物学行为上均与原发性SCLC存在显著差异。首先，原发性SCLC以胚系或早期体细胞*RB1/TP53*双失活为普遍特征，常伴随WGD等经典SCLC分子改变，常见于中老年男性吸烟者；而转化型SCLC多由EGFR驱动突变阳性的NSCLC在治疗选择压力下继发获得性*RB1/TP53*共失活以驱动谱系转化，多见于非吸烟的女性。其次，转化型SCLC因兼具腺癌与神经内分泌表型，对传统化疗的敏感性普遍低于原发性SCLC，且对既往EGFR-TKIs耐药，治疗选择更为有限。最后，转化型SCLC是肿瘤经治疗压力后发生的谱系可塑性改变，恶性程度更高、进展更迅速，临床预后显著劣于原发性SCLC。此病例为罕见的“双驱动”基因异常合并SCLC转化的复杂耐药表型，又与经典的单纯SCLC转化型病例存在明显区别。在分子特征上，该患者的多重耐药格局提示肿瘤在靶向治疗及放疗联合压力下可发生更为复杂的平行克隆进化。

针对SCLC转化的患者，临床推荐参照原发性SCLC的标准一线治疗方案，即依托泊苷联合铂类药物，客观缓解率可达50%以上，中位OS达9-11个月^[14]^。也有研究^[15]^显示SCLC转化的患者采用单纯化疗或化疗联合免疫治疗亦能取得更优的疗效。ALK-TKIs主要针对ALK融合阳性的NSCLC有效，对转化型SCLC的疗效缺乏明确获益证据。本病例采用SCLC标准治疗策略，即口服依托泊苷胶囊进行化疗，符合临床诊疗原则。该患者因不良反应停用化疗后转为免疫联合抗血管生成药物维持治疗（阿得贝利单抗+安罗替尼），PFS达到8个月。转化型SCLC常伴随较高的TMB、基因组不稳定性及肿瘤微环境异常等特征^[11]^，免疫检查点抑制剂联合抗血管生成治疗，可能通过促进肿瘤血管正常化、缓解肿瘤免疫抑制微环境、提升效应T细胞浸润水平等机制，发挥协同抗肿瘤作用。

综上所述，本文报道1例EGFR突变型肺腺癌经一线奥希替尼治疗后并存SCLC转化与ALK融合的罕见病例。尽管存在单病例报道、穿刺活检限制基线组织学完整性等局限性，本研究凸显了在此类患者治疗过程中肿瘤分子图谱的动态变化，以及个体化治疗的重要性。
